# Crystal structure of anilazine

**DOI:** 10.1107/S160053681401647X

**Published:** 2014-08-01

**Authors:** Youngeun Jeon, Jineun Kim, Gihang Kang, Tae Ho Kim

**Affiliations:** a Department of Chemistry and Research Institute of Natural Sciences, Gyeongsang National University, Jinju 660-701, Republic of Korea

**Keywords:** crystal structure, anilazine, 1,3,5-triazin-2-amine, triazine fungicides, hydrogen bonding, Cl⋯Cl contacts, weak π–π inter­actions

## Abstract

The title compound [systematic name: 4,6-di­chloro-*N*-(2-chloro­phen­yl)-1,3,5-triazin-2-amine], C_9_H_5_Cl_3_N_4_, is a triazine fungicide. The dihedral angle between the planes of the triazine and benzene rings is 4.04 (8)°. In the crystal, two weak C—H⋯N hydrogen bonds and short Cl⋯Cl contacts [3.4222 (4) Å] link adjacent mol­ecules, forming two-dimensional networks parellel to the (112) plane. The planes are linked by weak inter­molecular π–π inter­actions [3.6428 (5) and 3.6490 (5) Å], resulting in a three-dimensional architecture.

## Related literature   

For information on the fungicidal properties of the title compound, see: Couture & Sutton (1978 ▶); Mercan & Inam (2008 ▶). For a related structure, see: Zeng *et al.* (2005 ▶)
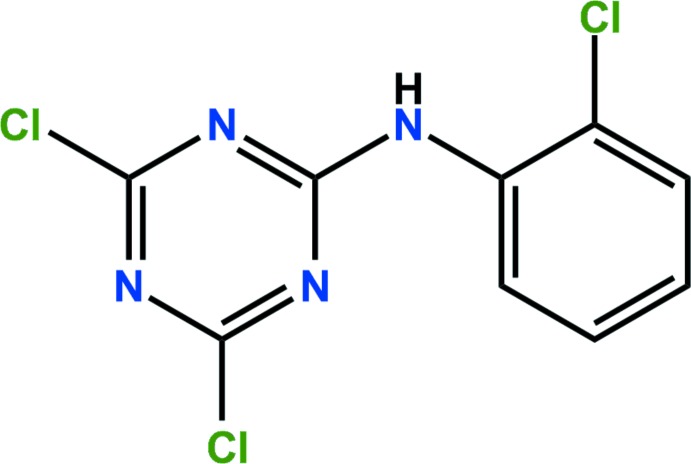


## Experimental   

### Crystal data  

C_9_H_5_Cl_3_N_4_
*M* *_r_* = 275.52
Triclinic, 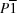

*a* = 7.2491 (9) Å
*b* = 7.9910 (9) Å
*c* = 10.5039 (13) Å
α = 111.954 (6)°
β = 106.411 (6)°
γ = 90.111 (6)°
*V* = 537.38 (11) Å^3^
*Z* = 2
Mo *K*α radiation
μ = 0.83 mm^−1^
*T* = 173 K
0.76 × 0.23 × 0.12 mm

### Data collection  

Bruker APEXII CCD diffractometer
Absorption correction: multi-scan (*SADABS*; Bruker, 2009 ▶) *T*_min_ = 0.573, *T*_max_ = 0.907
8699 measured reflections
2086 independent reflections
1877 reflections with *I* > 2σ(*I*)
*R*_int_ = 0.021

### Refinement  

*R*[*F* ^2^ > 2σ(*F* ^2^)] = 0.034
*wR*(*F* ^2^) = 0.087
*S* = 1.07
2086 reflections
145 parameters
H-atom parameters constrained
Δρ_max_ = 0.30 e Å^−3^
Δρ_min_ = −0.23 e Å^−3^

### 

Data collection: *APEX2* (Bruker, 2009 ▶); cell refinement: *SAINT* (Bruker, 2009 ▶); data reduction: *SAINT*; program(s) used to solve structure: *SHELXTL* (Sheldrick, 2008 ▶); program(s) used to refine structure: *SHELXTL*; molecular graphics: *DIAMOND* (Brandenburg, 2010 ▶); software used to prepare material for publication: *SHELXTL*.

## Supplementary Material

Crystal structure: contains datablock(s) global, I. DOI: 10.1107/S160053681401647X/hg5398sup1.cif

Structure factors: contains datablock(s) I. DOI: 10.1107/S160053681401647X/hg5398Isup2.hkl

Supporting information file. DOI: 10.1107/S160053681401647X/hg5398Isup3.cml

**Fig. 1.**
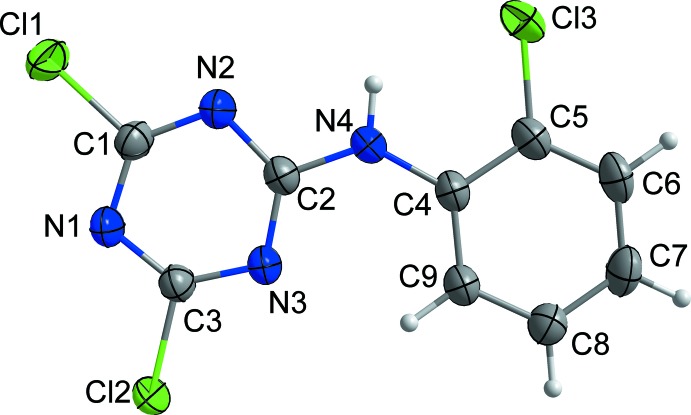
The molecular structure of the title compound. Displacement ellipsoids are drawn at the 50% probability level. H atoms are shown as small spheres of arbitrary radius.

. DOI: 10.1107/S160053681401647X/hg5398fig1.tif
The mol­ecular structure of the title compound. Displacement ellipsoids are drawn at the 50% probability level. H atoms are shown as small spheres of arbitrary radius.

**Fig. 2.**
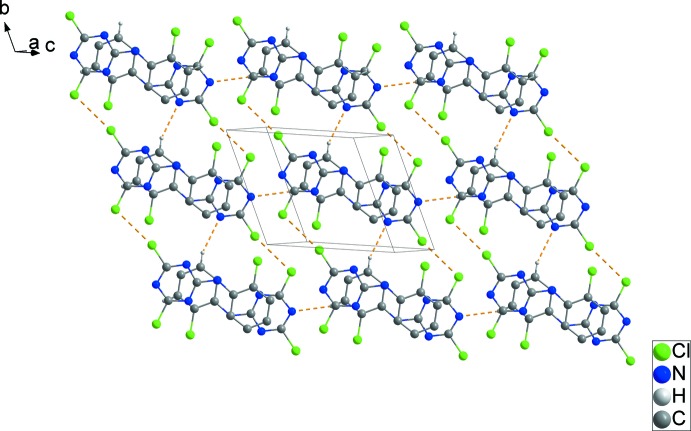
Packing diagram of the title compound with C—H···N hydrogen bonds and short Cl···Cl contacts shown as dashed lines. H atoms bonded to C atoms have been omitted for clarity, except H atoms of hydrogen bonds.

. DOI: 10.1107/S160053681401647X/hg5398fig2.tif
Packing diagram of the title compound with C—H⋯N hydrogen bonds and short Cl⋯Cl contacts shown as dashed lines. H atoms bonded to C atoms have been omitted for clarity, except H atoms of hydrogen bonds.

CCDC reference: 1014189

Additional supporting information:  crystallographic information; 3D view; checkCIF report

## Figures and Tables

**Table 1:** Hydrogen-bond geometry (Å, °)

*D*—H⋯*A*	*D*—H	H⋯*A*	*D*⋯*A*	*D*—H⋯*A*
C6—H6⋯N1^i^	0.95	2.64	3.568 (3)	165
C8—H8⋯N2^ii^	0.95	2.71	3.605 (3)	158

Symmetry codes: (i) 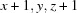; (ii) 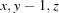.

## supplementary crystallographic information

### S1. Comment

Anilazine, C_9_H_5_Cl_3_N_4_, is a triazine fungicide used in controlling fungus diseases which attack lawns and turf, cereals, coffee, and a wide variety of vegetables and other crops. It is also used for the control of potato and tomato leaf spots (Couture & Sutton, 1978, Mercan & Inam, 2008). However, until now its crystal structure has not been reported.

In this compound (Fig. 1), the dihedral angle between dichloro phenyl ring and chlorophenyl phenyl ring is 4.04 (8)°. All bond lengths and bond angles are normal and comparable to those observed in a similar triazine fungicide structure, *N*-(4,6-Dichloro-1,3,5-triazin-2-yl)aniline(diclofop methyl) (Zeng *et al.*, 2005).

In the crystal structure (Fig. 2), two C—H··· N hydrogen bonds are observed (Table 1), forming two-dimensional networks parelle to (112) plane. The planes are linked by weak intermolecular π–π interactions [*Cg*1···*Cg*^iii^ , 3.6428 (5) and *Cg*1···*Cg*2^iv^, 3.6490 (5) Å], resulting in a three-dimensional architecture (*Cg*1 and *Cg*2 are the centroid of the N1···C3 and C4···C9 rings, respectively). In addition, a short Cl···Cl contact [Cl1···Cl1^v^, 3.4222 (4) Å] is present [for symmetry codes: (iii), -*x* + 1, -*y+*1, -*z* + 1, (iv), -*x* + 2, -*y+*1, -*z* + 1, and (v), *x*, *y+*1, -*z*].

### S2. Experimental

The title compound was purchased from the Dr. Ehrenstorfer GmbH Company. Slow evaporation of a solution in CHCl_3_ gave single crystals suitable for X-ray analysis.

### S3. Refinement

All H-atoms were positioned geometrically and refined using a riding model with d(C—H) = 0.95 Å, *U*_iso_ = 1.2*U*_eq_(C) for aromatic C—H groups.

### Crystal data

**Table:**

C_9_H_5_Cl_3_N_4_	*Z* = 2
*M**_r_* = 275.52	*F*(000) = 276
Triclinic, *P*1	*D*_x_ = 1.703 Mg m^−^^3^
Hall symbol: -P 1	Mo *K*α radiation, λ = 0.71073 Å
*a* = 7.2491 (9) Å	Cell parameters from 4910 reflections
*b* = 7.9910 (9) Å	θ = 2.8–28.4°
*c* = 10.5039 (13) Å	µ = 0.83 mm^−^^1^
α = 111.954 (6)°	*T* = 173 K
β = 106.411 (6)°	Plate, colourless
γ = 90.111 (6)°	0.76 × 0.23 × 0.12 mm
*V* = 537.38 (11) Å^3^	

### Data collection

**Table:**

Bruker APEXII CCD diffractometer	2086 independent reflections
Radiation source: fine-focus sealed tube	1877 reflections with *I* > 2σ(*I*)
Graphite monochromator	*R*_int_ = 0.021
φ and ω scans	θ_max_ = 26.0°, θ_min_ = 2.2°
Absorption correction: multi-scan (*SADABS*; Bruker, 2009)	*h* = −8→8
*T*_min_ = 0.573, *T*_max_ = 0.907	*k* = −9→9
8699 measured reflections	*l* = −12→12

### Refinement

**Table:**

Refinement on *F*^2^	Primary atom site location: structure-invariant direct methods
Least-squares matrix: full	Secondary atom site location: difference Fourier map
*R*[*F*^2^ > 2σ(*F*^2^)] = 0.034	Hydrogen site location: inferred from neighbouring sites
*wR*(*F*^2^) = 0.087	H-atom parameters constrained
*S* = 1.07	*w* = 1/[σ^2^(*F*_o_^2^) + (0.0383*P*)^2^ + 0.3274*P*] where *P* = (*F*_o_^2^ + 2*F*_c_^2^)/3
2086 reflections	(Δ/σ)_max_ = 0.001
145 parameters	Δρ_max_ = 0.30 e Å^−^^3^
0 restraints	Δρ_min_ = −0.23 e Å^−^^3^

### Special details

**Table:**

Geometry. All e.s.d.'s (except the e.s.d. in the dihedral angle between two l.s. planes) are estimated using the full covariance matrix. The cell e.s.d.'s are taken into account individually in the estimation of e.s.d.'s in distances, angles and torsion angles; correlations between e.s.d.'s in cell parameters are only used when they are defined by crystal symmetry. An approximate (isotropic) treatment of cell e.s.d.'s is used for estimating e.s.d.'s involving l.s. planes.
Refinement. Refinement of *F*^2^ against ALL reflections. The weighted *R*-factor *wR* and goodness of fit *S* are based on *F*^2^, conventional *R*-factors *R* are based on *F*, with *F* set to zero for negative *F*^2^. The threshold expression of *F*^2^ > σ(*F*^2^) is used only for calculating *R*-factors(gt) *etc*. and is not relevant to the choice of reflections for refinement. *R*-factors based on *F*^2^ are statistically about twice as large as those based on *F*, and *R*- factors based on ALL data will be even larger.

### Fractional atomic coordinates and isotropic or equivalent isotropic displacement parameters (Å^2^)

**Table:**

	*x*	*y*	*z*	*U*_iso_*/*U*_eq_	
Cl1	0.47189 (10)	0.95710 (7)	0.24807 (6)	0.05066 (19)	
Cl2	0.39173 (9)	0.25864 (7)	0.08297 (5)	0.04410 (17)	
Cl3	1.07091 (9)	0.84421 (8)	0.85361 (6)	0.0551 (2)	
N1	0.4406 (2)	0.6089 (2)	0.17713 (17)	0.0349 (4)	
N2	0.6364 (3)	0.7946 (2)	0.41465 (18)	0.0352 (4)	
N3	0.5964 (2)	0.4720 (2)	0.33916 (17)	0.0316 (4)	
N4	0.7900 (3)	0.6655 (2)	0.56838 (18)	0.0347 (4)	
H4N	0.8274	0.7803	0.6264	0.042*	
C1	0.5218 (3)	0.7666 (3)	0.2854 (2)	0.0347 (5)	
C2	0.6700 (3)	0.6401 (3)	0.4365 (2)	0.0306 (4)	
C3	0.4865 (3)	0.4707 (3)	0.2148 (2)	0.0317 (4)	
C4	0.8659 (3)	0.5413 (3)	0.6295 (2)	0.0307 (4)	
C5	1.0003 (3)	0.6117 (3)	0.7676 (2)	0.0362 (5)	
C6	1.0775 (3)	0.5011 (3)	0.8385 (2)	0.0425 (5)	
H6	1.1661	0.5521	0.9332	0.051*	
C7	1.0244 (3)	0.3153 (3)	0.7702 (3)	0.0452 (6)	
H7	1.0767	0.2380	0.8181	0.054*	
C8	0.8961 (3)	0.2422 (3)	0.6332 (2)	0.0403 (5)	
H8	0.8619	0.1143	0.5862	0.048*	
C9	0.8157 (3)	0.3542 (3)	0.5627 (2)	0.0362 (5)	
H9	0.7260	0.3023	0.4684	0.043*	

### Atomic displacement parameters (Å^2^)

**Table:**

	*U*^11^	*U*^22^	*U*^33^	*U*^12^	*U*^13^	*U*^23^
Cl1	0.0751 (4)	0.0358 (3)	0.0427 (3)	0.0136 (3)	0.0125 (3)	0.0211 (2)
Cl2	0.0546 (4)	0.0341 (3)	0.0310 (3)	0.0005 (2)	−0.0021 (2)	0.0101 (2)
Cl3	0.0551 (4)	0.0449 (3)	0.0403 (3)	−0.0067 (3)	−0.0071 (3)	0.0055 (2)
N1	0.0384 (10)	0.0364 (9)	0.0292 (9)	0.0060 (7)	0.0056 (7)	0.0155 (7)
N2	0.0398 (10)	0.0328 (9)	0.0315 (9)	0.0042 (7)	0.0070 (8)	0.0137 (7)
N3	0.0326 (9)	0.0330 (8)	0.0274 (8)	0.0040 (7)	0.0047 (7)	0.0131 (7)
N4	0.0389 (10)	0.0306 (8)	0.0269 (8)	0.0012 (7)	0.0015 (7)	0.0090 (7)
C1	0.0400 (12)	0.0339 (10)	0.0346 (11)	0.0075 (9)	0.0125 (9)	0.0173 (9)
C2	0.0301 (10)	0.0337 (10)	0.0270 (10)	0.0031 (8)	0.0074 (8)	0.0116 (8)
C3	0.0322 (10)	0.0333 (10)	0.0284 (10)	0.0040 (8)	0.0074 (8)	0.0120 (8)
C4	0.0278 (10)	0.0376 (10)	0.0257 (9)	0.0038 (8)	0.0061 (8)	0.0129 (8)
C5	0.0319 (11)	0.0419 (11)	0.0291 (10)	0.0005 (9)	0.0056 (9)	0.0102 (9)
C6	0.0330 (11)	0.0604 (14)	0.0309 (11)	0.0038 (10)	0.0017 (9)	0.0203 (10)
C7	0.0400 (13)	0.0568 (14)	0.0462 (13)	0.0113 (11)	0.0074 (10)	0.0322 (11)
C8	0.0416 (12)	0.0390 (11)	0.0413 (12)	0.0052 (9)	0.0096 (10)	0.0190 (10)
C9	0.0360 (11)	0.0376 (11)	0.0306 (11)	0.0028 (9)	0.0031 (9)	0.0135 (9)

### Geometric parameters (Å, º)

**Table:**

Cl1—C1	1.724 (2)	N4—H4N	0.8800
Cl2—C3	1.722 (2)	C4—C9	1.389 (3)
Cl3—C5	1.735 (2)	C4—C5	1.399 (3)
N1—C3	1.319 (3)	C5—C6	1.382 (3)
N1—C1	1.332 (3)	C6—C7	1.383 (3)
N2—C1	1.311 (3)	C6—H6	0.9500
N2—C2	1.348 (3)	C7—C8	1.375 (3)
N3—C3	1.320 (2)	C7—H7	0.9500
N3—C2	1.340 (2)	C8—C9	1.394 (3)
N4—C2	1.351 (2)	C8—H8	0.9500
N4—C4	1.407 (2)	C9—H9	0.9500
			
C3—N1—C1	111.18 (17)	C5—C4—N4	117.55 (18)
C1—N2—C2	113.30 (17)	C6—C5—C4	121.7 (2)
C3—N3—C2	112.78 (17)	C6—C5—Cl3	118.75 (16)
C2—N4—C4	131.55 (17)	C4—C5—Cl3	119.51 (16)
C2—N4—H4N	114.2	C5—C6—C7	119.3 (2)
C4—N4—H4N	114.2	C5—C6—H6	120.3
N2—C1—N1	128.42 (18)	C7—C6—H6	120.3
N2—C1—Cl1	116.36 (15)	C8—C7—C6	120.1 (2)
N1—C1—Cl1	115.21 (15)	C8—C7—H7	120.0
N3—C2—N2	125.21 (17)	C6—C7—H7	120.0
N3—C2—N4	120.43 (17)	C7—C8—C9	120.6 (2)
N2—C2—N4	114.35 (17)	C7—C8—H8	119.7
N1—C3—N3	129.10 (19)	C9—C8—H8	119.7
N1—C3—Cl2	115.58 (15)	C4—C9—C8	120.32 (19)
N3—C3—Cl2	115.32 (15)	C4—C9—H9	119.8
C9—C4—C5	117.95 (18)	C8—C9—H9	119.8
C9—C4—N4	124.50 (18)		
			
C2—N2—C1—N1	−1.0 (3)	C2—N4—C4—C9	−5.2 (4)
C2—N2—C1—Cl1	−179.78 (15)	C2—N4—C4—C5	175.2 (2)
C3—N1—C1—N2	1.1 (3)	C9—C4—C5—C6	−1.9 (3)
C3—N1—C1—Cl1	179.87 (15)	N4—C4—C5—C6	177.6 (2)
C3—N3—C2—N2	1.0 (3)	C9—C4—C5—Cl3	178.72 (16)
C3—N3—C2—N4	−178.20 (18)	N4—C4—C5—Cl3	−1.7 (3)
C1—N2—C2—N3	−0.2 (3)	C4—C5—C6—C7	1.5 (3)
C1—N2—C2—N4	179.11 (18)	Cl3—C5—C6—C7	−179.08 (18)
C4—N4—C2—N3	2.5 (3)	C5—C6—C7—C8	0.0 (4)
C4—N4—C2—N2	−176.9 (2)	C6—C7—C8—C9	−1.1 (4)
C1—N1—C3—N3	0.0 (3)	C5—C4—C9—C8	0.8 (3)
C1—N1—C3—Cl2	−179.43 (15)	N4—C4—C9—C8	−178.8 (2)
C2—N3—C3—N1	−1.0 (3)	C7—C8—C9—C4	0.7 (3)
C2—N3—C3—Cl2	178.47 (14)		

### Hydrogen-bond geometry (Å, º)

**Table:**

*D*—H···*A*	*D*—H	H···*A*	*D*···*A*	*D*—H···*A*
C6—H6···N1^i^	0.95	2.64	3.568 (3)	165
C8—H8···N2^ii^	0.95	2.71	3.605 (3)	158

Symmetry codes: (i) *x*+1, *y*, *z*+1; (ii) *x*, *y*−1, *z*.
