# Supplementary figures and images for: Crystal structure of anilazine
Source: Acta Crystallogr Sect E Struct Rep Online. 2014 Aug 1;70(Pt 9):o923. doi: 10.1107/S160053681401647X (PMC4186163; doi:10.1107/S160053681401647X)

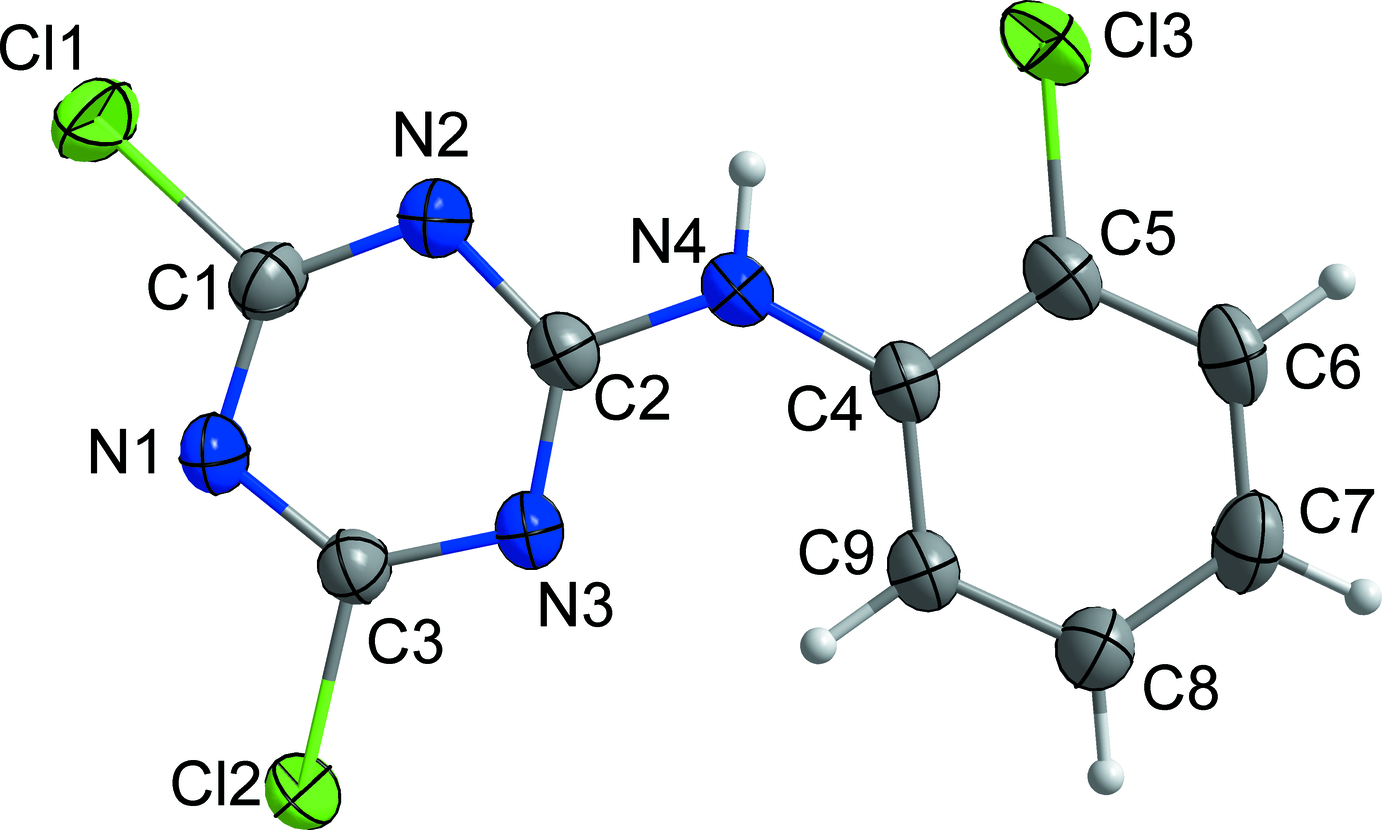

Supplement: Supplementary file 4 [file e-70-0o923-fig1.tif]

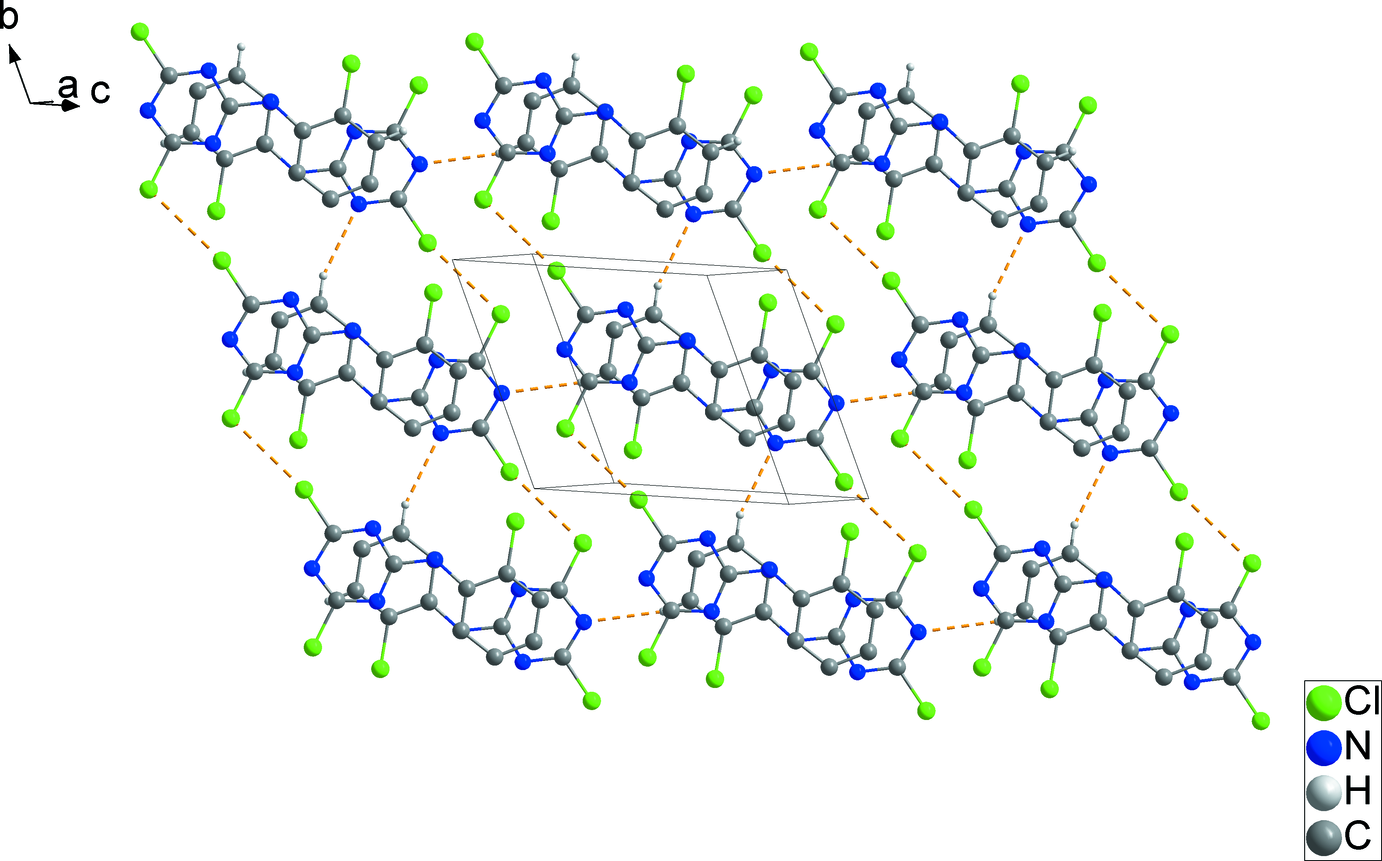

Supplement: Supplementary file 5 [file e-70-0o923-fig2.tif]
